# Sickle Cell Disease and COVID-19 Infection: Importance of COVID-19 Testing and Approach to Management

**DOI:** 10.7759/cureus.23604

**Published:** 2022-03-29

**Authors:** Zaryab Umar, Usman Ilyas, Nso Nso

**Affiliations:** 1 Internal Medicine, Icahn School of Medicine at Mount Sinai, Queens Hospital Center, New York, USA; 2 Department of Medicine, Ichan School of Medicine at Mount Sinai, NYC Health and Hospitals, New York, USA

**Keywords:** covid-19 pandemic, acute chest syndrome, sickle cell crisis, sickle cell disease, covid 19

## Abstract

Sickle cell disease is an autosomal recessive disorder resulting in the substitution of CTG by CAG in the sixth codon of the beta-globin gene. As a result of this, the hydrophilic glutamic acid residue is replaced by hydrophobic valine residue, leading to the formation of hemoglobin tetramer HBS. This alteration in the beta-globin chain makes the red blood cells prone to sickling, especially in the presence of risk factors such as stress, hypoxia, and infection. These sickled red blood cells have the tendency to adhere to the endothelium and lead to vessel occlusion and distal tissue ischemia. The recent coronavirus disease 2019 (COVID-19) outbreak has impacted millions across the globe, putting individuals with co-morbidities at particularly high risk, and patients with sickle cell disease are no exception.

We present the case of a 47-year-old African American male presenting to the emergency department with subjective fevers and a two-day history of pain in the arms, legs, and chest. A diagnosed case of sickle cell disease, the patient was on hydromorphone for pain management but ran out of his medications a few weeks before presentation. On examination, the patient was saturating well with mild tenderness upon palpation of the arms, legs, and chest. On complete blood count, the patient had a hemoglobin of 11.3 g/dL and a white cell count of 13.1 x10(3)/mcL. The patient had a normal mean corpuscular volume with reticulocytosis, hypochromia, ovalocytosis, poikilocytosis, polychromasia, and target cells. Severe acute respiratory syndrome coronavirus 2 (SARS-CoV-2) polymerase chain reaction (PCR) was positive. The chest X-ray did not reveal any significant findings. He was admitted to the medicine floor for the management of sickle cell crisis and was placed under airborne and droplet precautions. The patient was started on hydromorphone for pain management and intravenous fluid hydration. On the second day of admission, the patient reported increasing shortness of breath. He was saturating 90% on room air and 94% on 2 liters of supplemental oxygen. The white blood cell count increased to 18.42 x10(3)/mcL and the chest X-ray revealed reticular densities with patchy alveolar opacities in the left lung. Given the decline in respiratory status, the patient was started on remdesivir. Over the course of his hospital stay, the patient's pain and respiratory status improved, with the patient saturating 97% on room air. He was discharged home with instructions to follow isolation precautions for at least two weeks, folic acid, and adequate pain management. An appointment was also scheduled for the patient to follow with a sickle cell nurse practitioner upon discharge.

## Introduction

The coronavirus disease 2019 (COVID-19) exhibits a variable presentation that depends on the clinical and demographic characteristics of the infected patients [1]. Recent studies reveal fatal complications in 18.5% of patients with severe acute respiratory syndrome coronavirus 2 (SARS-CoV-2), which predominantly triggers COVID-19 [2]. In addition, more than 80% of the patients with COVID-19 experience mild-to-moderate symptoms that impact their prognostic outcomes. The preexisting morbidities and comorbidities also contribute to the severity of clinical manifestations in COVID-19 cases. The COVID-19 infection potentiates vaso-occlusive crises in patients with a history of sickle cell disease (SCD). The preexisting SCD further adds to the risk and incidence of multiple comorbidities, including pulmonary hypertension/chronic lung disease and renal insufficiency. The chronic lung damage caused by thrombo-inflammation due to COVID-19 aggravates the SCD complications and increases the risk of mortality [3]. The acute vaso-occlusive crises due to SCD in COVID-19 cases increase the probability of pulmonary embolism and acute chest syndrome. The hypercoagulable state of SCD-affected COVID-19 patients has a 3.5-fold risk of pulmonary emboli compared to the COVID-19 patients without SCD [4]. The findings in the contemporary literature do not provide concrete evidence regarding the attribution of COVID-19 to vaso-occlusive crises in patients with sickle cell anemia (SCA) [5]. Clinical studies reveal the absence of COVID-19-related symptomatology and signs in the majority of patients with vaso-occlusive crises and a positive RT-PCR finding. In addition, the unique COVID-19 pneumonia presentation with a lack of superinfection, mucoid impactions, and centrilobular nodules rarely correlates with vaso-occlusive crises in SCD patients [6]. This case study presents a reverse transcription-polymerase chain reaction (RT-PCR)-positive patient who developed respiratory compromise despite the absence of COVID-19-related symptoms.

## Case presentation

**Figure 1:**
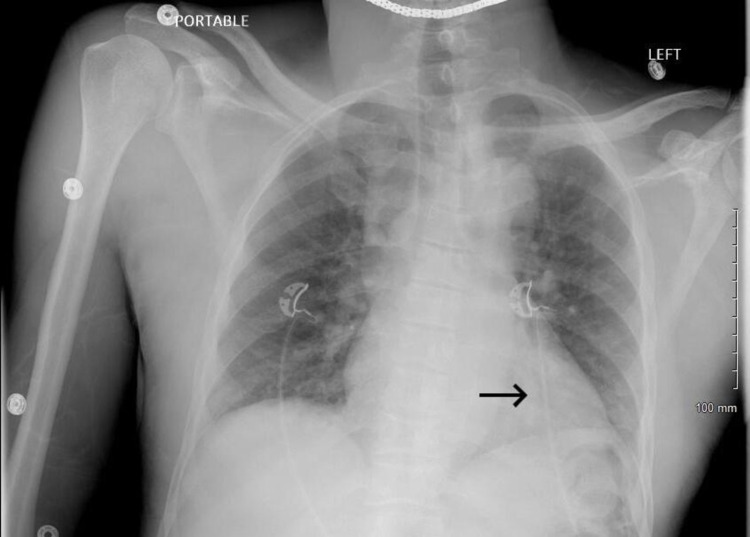
Chest X-ray showing reticular densities with patchy alveolar opacities in the left lung base/retro cardiac region (suggesting developing pneumonia)

**Figure 2:**
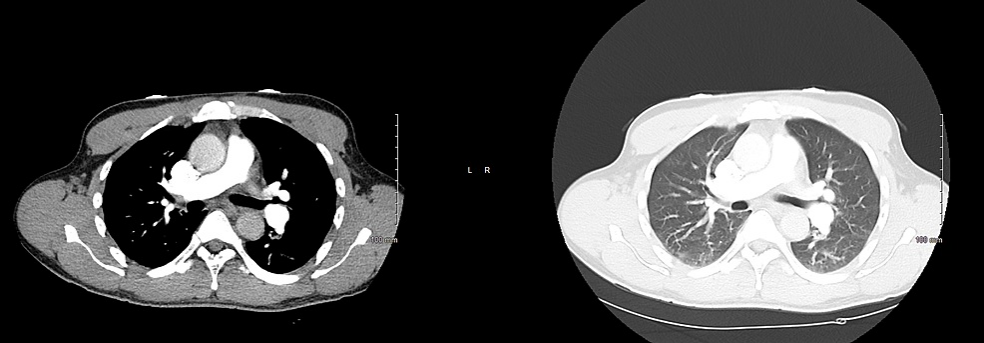
CT angiogram of the chest with no findings suggestive of pulmonary embolism

We report a 47-year-old African American male who presented to the emergency department with pain in his arms, legs, and chest for two days in duration. The patient was diagnosed case of sickle cell anemia with a history of two exchange transfusions. The patient also reported subjective fevers that started one day after the onset of pain. He had been prescribed Dilaudid for his pain but ran out of his pain medications a few weeks before presentation. The patient denied any shortness of breath, headaches, dysuria, and hematuria. On examination, the patient had mild tenderness upon palpation of the arms, legs, and chest. In the emergency department, the patient was saturating at 97% on room air. On complete blood count, the patient had a hemoglobin of 11.3 g/dL and a white cell count of 13.1 x10(3)/mcL (Table 1). The patient had a normal mean corpuscular volume (MCV) with reticulocytosis, hypochromia, ovalocytosis, poikilocytosis, polychromasia, and target cells (Table 2). His total bilirubin was 2.3 mg/dL with normal direct bilirubin. The patient’s SARS-CoV-2 PCR was positive. The chest X-ray did not reveal any significant findings and CT angiography chest with contrast do not reveal any central filling defect within the pulmonary arteries to suggest pulmonary embolism (Figures 1-2).

**Table 1:** Lab values obtained at the time of patient's initial presentation and Day 2 and Day 7 of admission MCV: mean corpuscular volume; MCH: mean corpuscular hemoglobin; MCHC: mean corpuscular hemoglobin concentration

Lab name (Reference range and units)	On the day of admission	Day 2 of admission	Day 7 of admission
Hemoglobin (14-18 g/dL)	11.3	11.5	10.3
Hematocrit (42.0-52.0%)	31.3	31.8	28.2
MCV (fL)	88.2	87.8	87.6
MCH (pg)	31.8	31.8	32.0
MCHC (g/dL)	36.1	36.2	36.5
WBC (4.80-10.80 x10(3)/mcL	13.10	18.42	20.08
Reticulocyte %	4.33	-	-

**Table 2:** Manual differential obtained at the time of admission

Manual differential on the day of admission	
Anisocytosis	Slight
Poikilocytosis	Moderate
Polychromasia	Slight
Target cell	Moderate

The patient was admitted to the medicine floor for the management of the sickle cell crisis and COVID-19 infection under airborne and droplet precautions. The patient was started on hydromorphone 2 mg every six hours as needed for the pain and half normal saline for hydration. Since the patient had good oxygen saturation on room air, steroids and remdesivir were not started. The patient was started on azithromycin.

On the second day of admission, the patient reported increased pain and new-onset shortness of breath. His WBC count increased to 18.42 x10(3)/mcL from an initial value of 13.10 x10(3)/mcL with the patient’s oxygen saturation being 90% on room air and 94% on 2 liters of supplemental oxygen (Table 1). D-dimers obtained were negative. Chest X-ray revealed reticular densities with patchy alveolar opacities in the left lung (Figure 1). For pain management, the hydromorphone regimen was altered to 2 mg every six hours standing, 1 mg every three hours PRN, and ketorolac 30 mg between hydromorphone doses. The patient was also started on remdesivir 100 mg daily for five days given the decline in respiratory status.

During the seven-day course of the hospital stay, the patient’s pain remained well-controlled. Hydromorphone 2 mg every six hours standing was reduced to 1 mg before switching to 4 mg oral every four hours, and Toradol was eventually discontinued. Gentle hydration was continued throughout. The patient’s respiratory status improved as well, with the patient saturating at 97% on room air. He was discharged home with instructions to follow isolation precautions for at least two weeks, folic acid, and adequate pain management. An appointment was also scheduled for the patient to follow with a sickle cell nurse practitioner upon discharge.

## Discussion

Sickle cell disease (SCD) is an autosomal recessive disorder that impacts the health and wellness of 100,000 individuals across the United States. In addition, SCD develops in African American people at birth with an incident ratio of 1:365 [7] and in Hispanic-American individuals with an incident ratio of 1: 16,300. The sickle cell trait affects Black children with an incident ratio of 1:13. A homozygous mutation in the beta-S allele on chromosome 11p15.5 triggers the development of SCD [8]. The sixth codon of the beta-globin gene undergoes substitution of GTG from GAG with a single nucleotide polymorphism dbSNP Rs334(T; T) difference from the wild-type beta allele. The erythrocytes of SCD patients contain a mutated hemoglobin tetramer HbS (α2βs2) developed by hydrophobic valine residue substitution from the hydrophilic glutamic acid residue. The different types of SCD develop from the inheritance or coinheritance of βS mutation (HbSS) with numerous genetic mutations attributing to β-thalassemia allele (HbS/β-thal0 or HbS/β-thal+), βE (HbSE), βO (HbSO/Arab), βD (HbSD), and βC (HbSC) [8]. The beta-globin chain mutations potentially increase the risk of red blood cell sickling under the impact of stress factors, including, infection, stress, weather changes/cold exposure, dehydration, and hypoxia. The erythrocyte membrane distortion and cellular rigidity under the impact of the HbS polymer further aggravate the sickling process that increases the risk and incidence of premature hemolysis, rheology impairment, and cellular energetic failure. The sickling process triggers the production of adhesion molecules that facilitate the attachment of red blood cells to the endothelium, thereby inducing distal tissue hypoxia and vaso-occlusion [9]. The perfusion injury by free radicals attributes to the accumulation of inflammatory mediators potentiated by red blood cell sickling and HbS polymers. A recent retrospective study by Shah et al. revealed a vaso-occlusive crises rate of 53.91 and 142.90 per 100 person-years in the setting of SCD [10]. The findings also indicated a high incidence of acute complications, including pulmonary hypertension, splenic sequestration, pulmonary embolism, stroke, and acute chest syndrome in patients with SCD. They also confirmed a high mortality predisposition in SCD scenarios based on vaso-occlusive crises (HR: 1.56, 95% CI 1.19-2.05). The analysis by Nicolas et al. confirmed a 0.7 per person-year rate of vaso-occlusive crises in adult patients with SCD [11]. 

The public health emergency across the globe due to COVID-19 is attributed to its rapid human-to-human transmission. In addition, factors including asymptomatic carriers, 41% hospital-based transmission, and lack of a standard treatment aggravated the rapid progression of the COVID-19 pandemic [12]. Patients with a history of SCD experience a high risk of fatal infections and respiratory complications after contracting SARS-CoV-2 [13]. A recent study by Panepinto et al. reported a 7% incidence of mortality and 69% hospitalization incidence in patients with SCD and COVID-19 [14]. The findings also revealed a 2% dialysis rate, 38% transfusion incidence, 6% ventilator requirement rate, and 11% ICU admission incidence in COVID-19-infected patients with SCD. These statistics are incomparable to age-related COVID-19 complications in the United States [15]. The extrapulmonary manifestations of SARS-CoV-2 include dermatologic complications, ocular conditions, neurological illnesses, ketosis, hyperglycemia, hepatocellular injury, gastrointestinal complications, acute kidney injury, acute coronary syndromes, arrhythmia, myocardial dysfunction, and thromboembolism [16]. The predominant theories for COVID-19-related cardiovascular manifestations advocate various factors attributing to the potentiation of the hypercoagulability phenomenon in the host cell lines. These factors include thrombotic microangiopathy [17], endothelial damage [18], reduced thrombin concentration [19], excessive intravascular platelet aggregation [20], and elevation in coagulation markers [21]. In addition, a cytokine storm (triggered by interleukin-6 hyperactivation) or macrophage activation syndrome triggers systemic hyper-inflammation, thereby inducing cardiolipotoxicity and vaso-occlusive crises in patients with COVID-19 [22-23]. The reportable symptoms of vaso-occlusive crises in COVID-19 scenarios include chest pain, flank pain, and leg pain [24]. The contemporary literature affirms vaso-occlusive (pain) crises as the commonly reported cause of emergency room visits and hospitalizations in patients with SCD [25]. The unpredictable and severe pain crises in SCD scenarios cause more than 90% of acute hospital admissions. The potential triggers of vaso-occlusive crises in SCD cases include hypoxia and infections that lead to cytokine release and coagulation dysfunction. The vaso-occlusive crises triggered by COVID-19 infection potentiate severe hemolysis that increases the risk of hospital admissions/readmissions and mortality [26]. These findings support the claim concerning the role of SARS-CoV-2 in triggering vaso-occlusive crises in patients with SCD.

The limited use of interleukin-6 receptor antagonists/immune-modulating drugs, patient age, and other unexplored pathophysiological mechanisms determine the progression of vaso-occlusive crises in COVID-19 scenarios [27]. Our case report is not devoid of limitations; however, it aims to unbox the key aspects of SCD-induced vaso-occlusive crises in patients with SARS-CoV-2. The COVID-19-positive SCD patients without fever, shortness of breath, and cough experience a high risk of fatal morbidities and mortality. The COVID-19 testing in asymptomatic SARS-CoV-2 patients with vaso-occlusive (pain) crises is therefore paramount to improving their treatment outcomes [28-29].

The patient (with COVID-19, SCD, and vaso-occlusive crises) we treated developed respiratory manifestations while the imaging results suggested pneumonia on the second day of hospital admission. We, therefore, recommend in-hospital management and close monitoring of respiratory distress in COVID-19-positive patients with vaso-occlusive crises. In addition, the packed red blood cell transfusion with a reduced threshold or borderline low hemoglobin levels is a putative approach to counter the high oxygen demand due to increased heme destruction by SARS-CoV-2 in patients with SCD [30]. The pharmacotherapy based on interleukin-6 inhibitor (tocilizumab) in COVID-19/SCD scenarios helps control cytokine storms and immune response dysregulation based on critical hypercytokinemia, neutrophilia, and lymphocytopenia [31]. A randomized study by Salama et al. indicated the potential of tocilizumab therapy in minimizing the composite incidence of death or mechanical ventilation without impacting the survival of patients with COVID-19 pneumonia [32]. These findings necessitate future research to testify to the therapeutic benefits of tocilizumab in COVID-19-positive patients with a history of SCD. 

## Conclusions

This case report supports the need for COVID-19 testing of all patients with SCD and suspected of vaso-occlusive crises. In addition, patients with COVID-19 and SCD require closed monitoring for respiratory distress to improve their prognosis, recovery, and scope of survival. Packed red blood cell transfusion at a low threshold minimizes the risk of respiratory compromise in COVID-19-positive SCD cases. Blood transfusion with/without exchange transfusion for sickle cell crisis and pharmacotherapy with steroids and remdesivir for COVID-19 infection minimize the risk of fatal complications and mortality. SCD potentiates acute multiorgan failure syndrome in patients with SARS-CoV-2 by dysregulating the hematological, immune, respiratory, neurological, and cardiovascular systems. However, proactive testing and low threshold treatment have the potential to improve health outcomes and minimize the disability-adjusted life years in patients with COVID-19 infection and SCD.
